# Bidirectional associations of accelerometer-assessed physical activity and sedentary time with physical function among older English adults: the EPIC-Norfolk cohort study

**DOI:** 10.1007/s10433-022-00733-y

**Published:** 2022-10-21

**Authors:** Dharani Yerrakalva, Samantha Hajna, Katrien Wijndaele, Paddy C. Dempsey, Kate Westgate, Nick Wareham, Simon J. Griffin, Soren Brage

**Affiliations:** 1 Department of Public Health and Primary Care, University of Cambridge, School of Clinical Medicine, Cambridge Institute of Public Health, Forvie Site, Robinson Way, Cambridge, CB2 0SR UK; 2 MRC Epidemiology Unit, University of Cambridge, School of Clinical Medicine, Cambridge, UK; 3 Physical Activity and Behavioural Epidemiology Laboratories, Baker Heart and Diabetes Institute, Melbourne, Australia; 4 Diabetes Research Centre, Leicester General Hospital, University of Leicester, Leicester, UK

**Keywords:** Physical activity, Sedentary time, Physical function, Older adults

## Abstract

To develop healthy ageing interventions, longitudinal associations between objectively assessed physical behaviours and physical function need to be better understood. We assessed associations between accelerometer-assessed total physical activity (PA), moderate-to-vigorous physical activity (MVPA), light physical activity (LPA), sedentary time and prolonged sedentary bout time, and clinically assessed physical function (grip strength, usual walking speed (UWS), chair stand speed) at two time-points in 3188 participants (≥ 60 years) of the EPIC-Norfolk study. Bidirectional associations were assessed using multivariable linear regression. Over an average of 6.1 years, baseline physical behaviours (greater total PA, MVPA and LPA, and less sedentary time) were associated with better subsequent walking and chair stand speed. Better baseline physical function was associated with better follow-up physical behaviours. There were no bidirectional associations between changes in physical behaviours and grip strength. Improvements in UWS were associated with improvements in all physical behaviours. Improvements in chair stand speed were associated with improvements in total PA, MVPA, and sedentary bout time. Improvements in physical behaviours were associated with improvements in UWS (3.1 cm/s/yr per 100 cpm/yr  total PA, 3.6 cm/s/yr per hr/day/yr MVPA, 2.5 cm/s/yr per hr/day/yr LPA, − 2.9 cm/s/yr per hour/day/yr sedentary time, and − 1.6 cm/s/yr per hr/day/yr prolonged sedentary bout time). Only improvements in total PA, MVPA and sedentary bout time were associated with improvements in chair stand speed. In conclusion, we found bidirectional associations between changes in some physical behaviours and physical function and between baseline physical behaviours and subsequent physical function, highlighting the importance of considering the full range of physical behaviours to promote healthy ageing.

**Supplementary Information:**

The online version contains supplementary material available at 10.1007/s10433-022-00733-y.

## Introduction

Healthy ageing is “the process of developing and maintaining the functional ability that enables wellbeing in older age”, and it involves “having the capabilities that enable all people to be and do what they have reason to value” (World Health Organization 2020). Functional ability is reliant on the mental and physical capacities of the individual (affected by presence of diseases, injuries and age-related changes) and the individual’s environment (the physical and psychological environments of home, community and broader society). Two important factors in the process of healthy ageing are an individual’s physical function and their physical behaviour. From herein, physical behaviour is used as an umbrella term to encapsulate both physical activity (PA) and sedentary time. Reduced physical function is associated with higher falls risk, loss of independence, multimorbidity and premature mortality (Smee et al. 2012; Vaughan et al. 2016; Calderón-Larrañaga et al. 2019; Wei et al. 2019). Concurrently, low levels of PA and high levels of sedentary time are associated with higher risk of falls, dementia, diabetes, cardiovascular and cancer morbidity (Thibaud et al. 2012; Krebs et al. 2018; Ekelund et al. 2019; Kouloutbani et al. 2019). PA and physical function both gradually decline over the life course, while sedentary time rises (Hajna et al. 2018; Takayanagi et al. 2019; Giné-Garriga et al. 2020). A significant proportion of UK older adults do not meet PA guidelines, with the number not meeting guidelines increasing with age (NHS Digital, 2019). To delay onset of age-related health declines and maintain healthy ageing, interventions aimed at promoting PA and physical function and reducing sedentary time are needed.

There are uncertainties concerning the relationship between sedentary time, PA and physical function which limit the ability to design effective interventions. Firstly, as almost all studies have used cross-sectional design, little is known about how change in physical behaviours over time influences change in physical function over time, and vice versa (Cooper et al. 2017; Metti et al. 2018; Hopkins 2019; Laddu et al. 2020). Secondly, few studies have utilised precise objective physical behaviour measures (Cooper et al. 2017; Metti et al. 2018; Laddu et al. 2020). Thirdly, of those cross-sectional studies that exist, the majority have focussed on moderate-to-vigorous activity (MVPA) only. Few studies have investigated sedentary time (Keevil et al. 2016; Foong et al. 2016; Dogra et al. 2017; van der Velde et al. 2017; Reid et al. 2018a; Edholm et al. 2019; Giné-Garriga et al. 2020), prolonged sedentary bout time (Liao et al. 2018) or light physical activity (LPA) (Edholm et al. 2019; Foong et al. 2016; Savikangas et al. 2020), with existing cross-sectional studies conflicting on whether an association exists. This is in spite of these behaviours being potentially easier to change than MVPA in older adults, given that change would take less physical effort.

To help elucidate relationships between PA, sedentary time and physical function and to inform development of interventions to promote healthy ageing, we aimed to quantify bidirectional associations between physical behaviours (total physical activity (TPA), MVPA, LPA, total sedentary time, prolonged sedentary bout time) and physical function (usual walking speed (UWS), handgrip strength and chair stand speed) using objective measures. Specifically, we aimed to estimate associations of baseline physical function with follow-up physical behaviours, of baseline physical behaviours with follow-up physical function, of change in physical function with change in physical behaviours, and of change in physical behaviours with change in physical function.

## Methods

We used data from the prospective EPIC-Norfolk cohort study (UK) which recruited 25,500 adults at baseline in the early 1990s. The cohort had broadly similar characteristics to other national population samples in terms of anthropometry, serum lipids, and blood pressure (Hayat et al. 2014) and has been followed up over five health-checks. Accelerometer measurement was carried out at the 3rd (2004–2011) and 4th (2012–2016) health checks (referred to as baseline and follow-up from here onwards), and therefore, data from these health-checks were included in this analysis. The baseline and follow-up assessments were attended by 7312 and 4992 participants aged ≥ 60, respectively. At the baseline health-check, there was a total of 176 accelerometers available. Prior to each visit, the available accelerometers were randomly assigned to individuals. In total, at the baseline health-check 3727 individuals were asked to wear an accelerometer and accepted. At the follow-up health-check, 4992 individuals were asked to wear an accelerometer and 4801 accepted. Those who refused to wear accelerometers at follow-up were socio-demographically similar to those who were included. Participants included in this analysis were slightly healthier than the general population in terms of blood pressure and body mass index (BMI) (Craig and Mindell 2007). In our sample, for men the average BMI was 27.1 and systolic blood pressure was 135.6, and in Health Survey for England, BMI was 27.5, and systolic blood pressure was 135.8. We included participants aged ≥ 60 at baseline with either valid baseline accelerometry and follow-up physical function data, or valid baseline physical function and follow-up accelerometry data.

### Accelerometry

At baseline, participants wore a uniaxial accelerometer (Actigraph GT1M™, USA). At follow-up, participants wore triaxial accelerometers (GT3X™ PLUS, USA). Actigraph GT3X PLUS generates raw (waveform) data within the software as uniaxial frequency-filtered counts, and this output is comparable to the output of the uniaxial accelerometers used at baseline. These accelerometer models are considered comparable in physical behaviour measurement (Robusto and Trost 2012; Ried-Larsen et al. 2012). Data from the two accelerometers were harmonised using previously described methods (Ried-Larsen et al. 2012). Participants were instructed to wear accelerometers on their right hip for seven days except when bathing, swimming or sleeping. Recorded activity was integrated into 60-s epochs (Edwardson and GORELY 2010). Variables derived from accelerometry data were TPA, time in MVPA, LPA, total sedentary time, and prolonged sedentary bouts (≥ 30 min). The intensity cut-offs used to define time (hours or minutes per day) in behaviours were < 100 counts per minute (cpm) for sedentary time, 100–808 cpm for LPA, and ≥ 809 cpm for MVPA (Gorman et al. 2014; Berkemeyer et al. 2016). TPA was calculated by total activity counts divided by wear time (counts/minute). Non-wear time was defined as continuous zero counts of ≥ 90 min (Mailey et al. 2014). To deal with overnight wear, we overlaid self-report sleep timings at epoch level for days with wear-time > 19 h and excluded data accordingly. Only participants with ≥ 4 days of valid wear-time (≥ ten hours of wear time each day) were included in analyses. We calculated the annual rate of change of accelerometer-assessed variables (e.g. min/day/year) as the difference between values at baseline and follow-up divided by follow-up time.

### Physical function

Physical function outcomes were grip strength, UWS, and chair stand speed. Measurements were taken by trained research staff following standardised protocols (Keevil et al. 2013). Grip strength (kg) was measured using a dynamometer (Smedley’s Dynamometer, Denmark) (Roberts et al. 2011). While standing, participants were asked to grip the dynamometer with maximum strength twice, alternating between hands, with the best effort recorded. To measure UWS (cm/second), participants were instructed to walk from standing start at usual walking speed for four metres, plus an additional metre to a line one metre beyond the finish line to ensure they maintained their usual speed all the way to the end of the course, with the average speed of walking the four-metre course across two attempts calculated ((400/1st attempt time to complete four-metre course) + (400/2nd attempt time to complete four-metre course)/2) (Kim et al. 2016). For chair stand speed (stands/minute), participants were asked to sit and stand five times (Bohannon et al. 2010). Speed was calculated as number of stands/minute (60 *(5/test completion time (s))). We calculated the annual rate of change as the difference between values at baseline and follow-up divided by follow-up time. Units for change in grip strength were kg/year, change in UWS were cm/second/year, and change in chair stand speed were stands/minute/year.

### Covariates

The covariates included were age, sex, ethnicity (white, other), occupational classification *(*Registrar*-*General's Social Classification which has five categories*;* I professional, II managerial/technical occupations III skilled occupations, IV partly skilled occupations and V unskilled occupations*),* job status (job vs no job), highest educational level (completed educational qualification at aged 16 (UK qualification is O level) or lower vs completed further education qualification at age 16–18 (UK qualification is A level) or higher), smoking status (never, former, current), chronic disease status (myocardial infarction, stroke, cancer or diabetes mellitus history) and body mass index (BMI). All these were assessed via self-completed questionnaire. BMI (kg/m^2^) was calculated based on weight and height measurements taken by trained research staff.

### Statistical analyses

To account for missing data, we performed complete case analyses. We assessed socio-demographic characteristics of those included and those excluded (due to missing data, and/or having less than 4 valid days of accelerometry wear-time). We undertook longitudinal analyses using multivariable linear regression models to estimate associations between (1) baseline physical function and follow-up physical behaviours, (2) baseline physical behaviours and follow-up physical function, (3) change in physical function and change in physical behaviours and (4) change in physical behaviours and change in physical function.

Analysis 3 examined the association between change in physical function (difference between baseline and follow-up physical function score divided by follow-up time, modelled as exposure) and change in physical behaviours (difference between baseline and follow-up physical behaviour scores, modelled as outcome). Analysis 4 examined the association between change in physical behaviours (modelled as exposure) and change in physical function (modelled as outcome).

We examined these associations across three models. Model 1 was adjusted for season (UK seasons can affect PA levels) (Stolwijk et al. 1999; Cepeda et al. 2018) and wear time at baseline and follow-up. Model 2 was the same as model 1 plus adjusted for age and sex. Model 3 was the same as Model 2 plus adjusted for potential socio-demographic confounders (job status, smoking status, occupational class, BMI, ethnicity, and chronic disease status). For the change in physical function and physical behaviour analyses (third and fourth analyses), adjustment for baseline physical behaviours and baseline physical function variables was added across all models.

We examined whether the associations between physical behaviours and physical function measures were modified by age < 70 versus ≥ 70 years old, to determine whether at this age cut-point the relationship changes, by adding an interaction term of age < 70 versus ≥ 70 years old. We carried out analyses to examine if accelerometer processing decisions (≥ 5 vs. ≥ 4 days of valid data and intensity cutpoint discriminating LPA and MVPA at 2020 cpm vs. 809 cpm) influenced results. We also examined the correlations between baseline and follow-up values of each PA and physical function measure to ascertain an understanding of stability of each measure, and therefore ability to detect change.

All analyses were conducted using STATA 15.0 (StataCorp, TX, USA).

## Results

Of the 3,673 older adults who had measurements at both baseline and follow-up health-checks, 485 individuals were excluded due to having < 4 valid days of accelerometry data (72 at baseline and 93 at follow-up excluded) or having missing variables (320 excluded), leaving a total of 3188 participants (87%). The average age of individuals was 69 years at baseline (SD = 6.0) (54% women) (Table 1). Participants who were included were similar in terms of average age (mean age of excluded 69.5, SD 6.5), sex, BMI, and education level to those excluded (Supplementary table 1). The median follow-up duration was 6.1 years (IQR = 4.7–6.9). The mean values of physical behaviour measures and physical function measures at baseline and follow-up are outlined in Table 2. For example, total sedentary time increased from baseline to follow-up by an average of 6.4 min/day/year (SD = 14.7) for women (0.9% increase) and 5.5 min/day/year (SD = 16.4) for men (1.0% increase) and MVPA decreased by an average of 3.6 min/day/year (SD = 8.7) for women (4.7% decrease) and 3.8 min/day/year (SD = 8.5) for men (4.8 decrease). Maximum grip strength decreased by 1.8% for females and 1.1% for males. UWS decreased by 0.2% for women and 0.3% for men. Chair stand speed increased by 1% for women and 1.5% for men.

**Table 1:** Frequency and percentage of baseline demographic and clinical characteristics (2006–2011) (*n* = 3188)

Characteristics	Frequency	Percent (%)
*Sex*		
Men	1453	45.6
Women	1735	54.4
*Ethnicity*		
White	3179	99.7
Other	9	0.3
*Occupational classification*		
Professional	287	9.0
Manager	1345	42.2
Skilled non-manual	467	14.7
Skilled manual	670	21.0
Semi-skilled	345	10.8
Non-skilled	74	2.3
*Employed*		
No	2402	75.4
Yes	786	24.6
*Further Education level*		
O-level or lower	1468	46.0
A-level or higher	1720	54.0
*Smoking Status*		
Current	107	3.4
Former	1462	46.2
Never	1599	50.4
*History of Chronic Disease*		
No	2706	84.9
Yes	482	15.1
*Body Mass Index (kg/m*^*2*^*)*		
< 25	1147	36.50
25–30	1459	45.8
30–35	441	13.8
> 35	125	3.9

**Table 2:** Mean physical behaviours and physical function values at baseline and follow-up

		Mean (SD) at baseline		Mean (SD) at follow-up		Mean annual change (SD)	
		Men	Women	Men	Women	Men	Women
Physical Function Variables	Maximum handgrip (kg)	39.7 (7.5)	24.8 (5.1)	36.7 (8.5)	22.3 (6.2)	− 0.44 (1.5)	− 0.45 (1.1)
	Usual walking speed (cm/s)	116 (24)	112 (23)	113 (25)	110 (25)	− 0.39 (4.6)	− 0.25(4.9)
	Chair stand speed (stands/min)	27.5 (8.2)	26.4 (8.0)	28.6 (7.8)	26.9 (7.6)	0.42 (1.7)	0.26 (1.5)
Physical Behaviour Variables	TPA (cpm)	251 (126)	251 (109)	233 (117)	220 (104)	− 9.2 (22.1)	− 8.9 (16.8)
	MVPA_809_ (min/day)	78.5 (48.2)	76.4(44.7)	70.4(46.0)	64.2(40.8)	− 3.8 (8.5)	− 3.6(8.7)
	LPA_809_ (min/day)	208 (54.5)	239 (54.2)	194.3 (55.0)	222(57.7)	− 4.0 (11.3)	− 4.0 (11.9)
	Total sedentary time (min/day)	585 (84)	542 (81)	600 (83)	568 (79)	+ 5.5(16.4)	+ 6.4 (14.7)
	Prolonged sedentary bout (min/day)	228 (101)	178(86)	259 (108)	215 (97)	+ 9.3 (19.8)	+ 9.0 (16.8)
	*Sensitivity Analysis (MVPA defined as* ≥ *2020 cpm, LPA defined as 100–2019 cpm)*						
	MVPA_2020_ (min/day)	23.0 (20.7)	18.3 (16.6)	21.4 (21.3)	16.5 (16.4)	− 1.0 (4.5)	− 0.8 (3.6)
	LPA_2020_ (min/day)	264 (78.4)	297 (76.1)	243 (78.7)	270 (78.4)	− 6.8 (14.6)	− 6.8(15.8)

TPA = total physical activity, MVPA = moderate-to-vigorous activity, LPA = light physical activity, SD = standard deviation

### Association of baseline physical function with follow-up physical behaviours

**Fig. 1:**
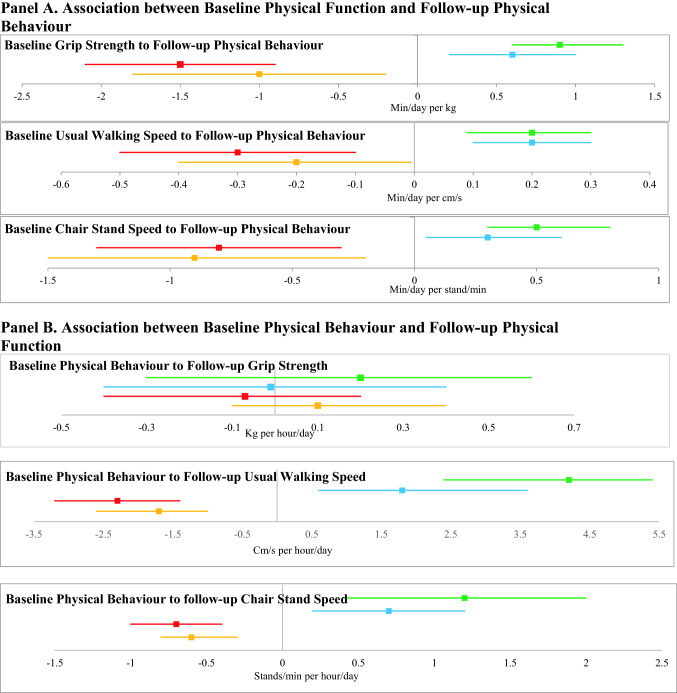

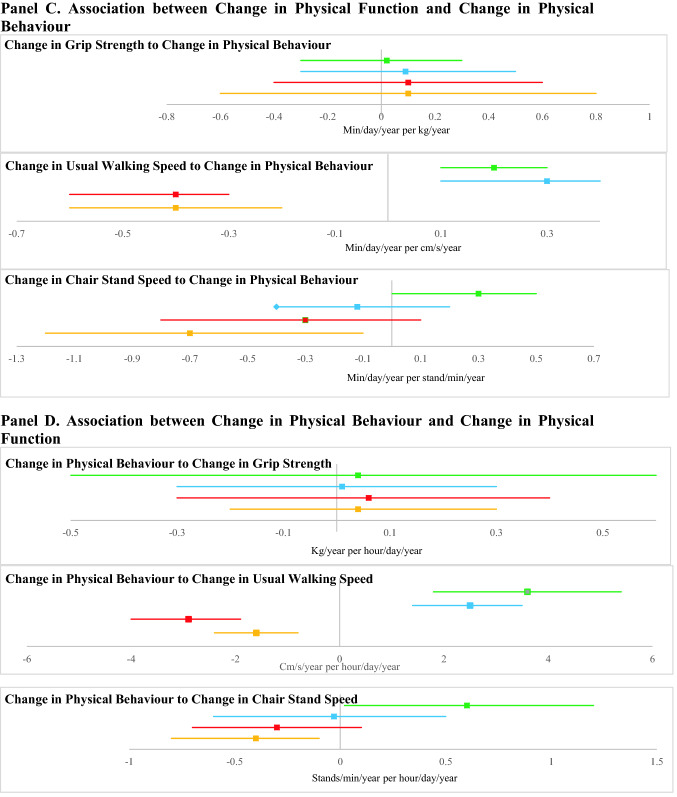
The bidirectional associations between physical function and physical behaviours. For all panels, MVPA is in green, LPA is in blue, ST is in red and Prolonged ST bouts is in orange. Beta is indicated by central square, 95% CI is indicated by the line. Baseline measures were taken between 2006 and 2011, and follow-up measures were taken between 2012 and 2016. Change in variables was from baseline to follow-up. In Panels A and B, results are from model 3. For Panel A, Beta is the number of mins/day of follow-up physical behaviours associated with each unit of the baseline physical function measure. For Panel B, Beta is the number of units of follow-up physical function measure associated with an hour/day of baseline physical behaviours. In Panels C and D, results are from model 3. For Panel C, Beta is the number of mins/day/year change in physical behaviour over follow-up associated with each unit/year change in the physical function measure. For Panel D, Beta is the number of units/year of change in physical function associated with an hour/day change in physical behaviour

Better baseline physical function was associated with higher levels of all follow-up PA measures (TPA, MVPA and LPA) and lower levels of sedentary time measures (Fig. 1 Panel A; Supplementary Table 2). Each extra kg of baseline grip strength was associated with 2.6 cpm higher follow-up TPA, 0.9 min/day higher follow-up MVPA, 0.6 min/day higher LPA, 1.5 min/day lower sedentary time, and 1.0 min/day lower prolonged sedentary bout time. Each extra cm/s of baseline UWS was associated with 0.6 cpm higher follow-up TPA, 0.2 min/day higher MVPA, 0.2 min/day higher LPA, 0.3 min/day lower sedentary time, and 0.2 min/day lower prolonged sedentary bout time. Each extra stand/min of baseline chair stand speed was associated with 1.5 cpm higher follow-up TPA, 0.5 min/day higher MVPA, 0.3 min/day higher LPA, 0.8 min/day lower sedentary time and 0.9 min/day lower prolonged sedentary bout time.

### Association of baseline physical behaviours with follow-up physical function

There was no association between any baseline physical behaviours measures and follow-up grip strength. Higher baseline TPA (2.9 cm/s per 100 cpm), MVPA (4.2 cm/s per hour/day) and LPA (1.8 cm/s per hour/day) and lower sedentary time (− 2.3 cm/s per hour/day) and prolonged sedentary bout time (− 1.7 cm/s per hour/day) were all associated with faster follow-up UWS (Fig. 1 Panel B; Supplementary Table 3). Higher baseline TPA (0.8 stands/min per 100 cpm), MVPA (1.2 stands/min per hour/day), and LPA (0.7 stands/min per hour/day) and lower baseline sedentary time (− 0.7 s stands/min per hour/day) and prolonged sedentary bout time (− 0.6 s stands/min per hour/day) were associated with faster follow-up chair stand speed.

### Association of change in physical function measures with change in physical behaviours measures

There were no significant associations between change in grip strength and any change in physical behaviour measures. Each cm/s/year improvement in UWS was associated with 0.6 cpm/year improvement in TPA, 0.2 min/day/year improvement in MVPA, 0.3 min/day/year improvement in LPA, 0.4 min/day/year reduction in sedentary time and 0.4 min/day/year reduction in prolonged sedentary bout time (Fig. 1 Panel C; Supplementary Table 4). Every stand/min/year improvement in chair stand speed was associated with 1.0 cpm/year improvement in TPA, 0.3 min/day/year improvement in MVPA and 0.7 min/day/year reduction in prolonged sedentary bout time. There was no association of change in chair stand speed with change in LPA or change in sedentary time.

### Association of change in physical behaviours measures with change in physical function

There were no significant associations between change in physical behaviour measures and change in grip strength. Greater improvements in TPA (3.1 cm/s/year per 100 cpm/year), MVPA (3.6 cm/s/year per hour/day/year) and LPA (2.5 cm/s/year per hour/day/year) and greater reductions in sedentary time (− 2.9 cm/s/year per hour/day/year) and prolonged sedentary bout time (− 1.6 cm/s/year per hour/day/year) were associated with improvements in UWS (Fig. 1 Panel D; Supplementary Table 5). Greater improvements in TPA (0.7 stands/min/year per 100 cpm/year) and in MVPA (0.6 stands/min/year per hour/day/year) and greater reductions in prolonged sedentary bout time (− 0.4 stands/min/year per hour/day/year) were associated with improvements in chair stand speed. There was no association of change in LPA or change in total sedentary time with change in chair stand speed.

In examining the stability of each variable, by examining correlation between baseline and follow-up value we found that baseline and follow-up grip strength were more strongly correlated (*R* = 0.83) than USW (*R* = 0.58) and chair stand speed (0.54) (Supplementary Table 6).

There was no significant interaction effect of age < 70 versus ≥ 70 years old on the associations of physical behaviour measures with physical function (data not shown). The results using different cut-points for MVPA and LPA discrimination (2020 vs. 809 cpm) showed similar results (Supplementary Tables 1–4). There were no important differences between our main results utilising ≥ 4 days of valid wear-time versus ≥ 5 days of valid wear-time (data not shown).

## Discussion

We here have confirmed bidirectional associations utilising longitudinal data and objective physical behaviour measures and have therefore addressed those uncertainties about the relationship between sedentary time, PA and physical function which we outlined earlier. We found that English older adults who had better baseline physical function, had better subsequent physical activity levels across all intensities and spent less time sedentary 6.1 years later. Furthermore, those with higher baseline physical activity levels across all intensities and who spent less time sedentary had better subsequent UWS and chair stand speed, but not grip strength.

Those who improved their UWS or chair stand speed over the follow-up, improved their TPA and MVPA and reduced their prolonged sedentary bout time. Further, those that improved their UWS, also improved their LPA and reduced their sedentary time. Those who improved their TPA and MVPA levels and reduced their prolonged sedentary bout time across follow-up had improvements in UWS and chair stand speed. Further, those who improved their LPA, and reduced their sedentary time showed improvements in UWS. However, those that changed their grip strength had no changes in any physical behaviour levels, and vice versa. Similarly, those that improved their chair stand speed did not improve their LPA and sedentary time, and vice versa.

### Context of findings with literature

A number of cross-sectional studies have showed significant positive associations between objectively measured MVPA and physical function in older adults, including UWS (Keevil et al. 2016; Foong et al. 2016; Edholm et al. 2019; Spartano et al. 2019; Savikangas et al. 2020; Lai et al. 2020; Westbury et al. 2021), grip strength (Keevil et al. 2016; Spartano et al. 2019; Lai et al. 2020), and chair stand speed (Keevil et al. 2016; Spartano et al. 2019), but research has largely neglected other activity intensities. We found only three cross-sectional studies examining associations between LPA and physical function, with two reporting a positive association (UWS (Savikangas et al. 2020) and dynamometer-measured knee extension strength (Foong et al. 2016)) and one reporting no association (UWS (Edholm et al. 2019)). Similarly, cross-sectional studies examining associations between sedentary time and physical function have shown mixed results with some showing negative associations (UWS (Keevil et al. 2016; van der Velde et al. 2017; Giné-Garriga et al. 2020), grip strength (Keevil et al. 2016; Dogra et al. 2017)) and some finding no association (UWS (Reid et al. 2018b; Edholm et al. 2019), chair stand speed (Keevil et al. 2016; Reid et al. 2018b), dynamometer-measured knee extension strength (Foong et al. 2016)). We found only one cross-sectional study examining the association of prolonged sedentary bout time with physical function, which found a negative association with both Timed-Up-and-Go Test and UWS (Liao et al. 2018). Cross-sectional studies examining the association between physical behaviours and physical function can only assume the direction of association. Taken together, the existing literature shows some evidence for an association between MVPA and physical function but not directionality of this relationship.

Our prospective work gives stronger evidence of temporal relationships between physical function and physical behaviours than the cross-sectional work outlined above. Our study is the first to examine longitudinal associations between baseline physical function and subsequent objectively assessed physical behaviours. We found only one study that has examined the longitudinal associations between baseline physical function and self-report PA (Cooper et al. 2017), which agreed with our findings. This study in UK Biobank (*n* = 6599, age >  = 60) found that baseline self-report PA was not associated with follow-up grip strength 4.5 years later, whereas baseline grip strength was associated with self-report PA at follow-up.

We know of no other studies which have examined the association between baseline TPA, LPA, sedentary time and prolonged sedentary bouts and follow-up physical function, and we are the second study to examine the associations between objectively measured baseline MVPA and follow-up physical function (Hopkins 2019). A US study of older adults (Hopkins 2019) (*n* = 687, mean age 63) found higher baseline MVPA (> 150 min/week vs. ≥ 150 min/week) was associated with faster subsequent 400-m walk test, but not 20-m UWS or chair stand speed 4 years later. This contrasts with our finding that higher baseline MVPA was associated with higher four-metre UWS and chair stand speed. This might have been for a number of reasons. Their study only examined two discrete categories of MVPA and had a smaller sample size, so may not have had the power to find a difference. Taken in the context of the literature described here, our findings newly report the bidirectional relationship between physical function and objectively assessed physical behaviours, support the cross-sectional literature regarding MVPA and extend the more scarce literature on the lesser studied behaviours of LPA and sedentary time.

We are also original in examining the bidirectional association between change in objectively measured physical behaviour measures and change in physical function. Three previous studies (Cooper et al. 2017; Metti et al. 2018; Laddu et al. 2020) have examined change in physical function and change in self-report PA, with mixed findings. A study in UK older adults (Cooper et al. 2017) found that those who maintained or increased PA did not change their grip strength (in agreement with our study), whereas those who increased their grip strength reported an additional 3.7 min/day (95% CI 0.20, 7.17) in PA over 4.5 years of follow-up (in contrast with our study). A US study (Laddu et al. 2020) classified men (mean age = 72, *n* = 3,865) into groups according to patterns of decline in self-reported PA (slow decline, intermediate decline and fast decline). They found that the slow decline group had the smallest decreases in UWS, chair stand speed and grip strength over seven year follow-up. This is in agreement with our results for UWS and chair stand speed but not grip strength. These two studies differ from ours in reporting the association of categories of change in self-report PA with physical function, and differed in their findings only on grip strength. The difference in our results may have been due to difference in measurement of PA (objective versus subjective, hip-worn accelerometers do not record upper body exercise or swimming and so studies using self-report PA may have been better in picking up upper body PA changes, examining total PA versus segments of the activity spectrum), or the treatment of change in PA as a categorical versus continuous variable.

Another US study (Metti et al. 2018) (mean age 77, *n* = 1,404) examined bidirectional associations between change in physical function (timed Up-and-Go task) and change in self-report PA. They found early change in physical function (years 0–5) was not associated with late change (years 5–9) in PA, and early change in PA was not associated with late change in physical function. Again, this difference in their findings compared to ours may have been due to PA measurement method (objective vs subjective), the difference in physical function measure used, the difference in change time period (we looked at change over the same period for both variables, whereas they looked at ‘early change’ and a ‘late change’ period), or the age of the population (our study population was younger). In summary, our findings using objective measures of physical behaviours are in partial agreement with the very limited existing literature that there is a bidirectional association between change in physical behaviours and change in physical function.

To provide clinical context to the observed effect sizes, we found that improvements in MVPA of the magnitude seen in RCTs (10 min/day/year (Chase 2015)) were associated with a 0.6 cm/s/year improvement in UWS and a 0.1 stands/min/year improvement in chair stand speed. Further, reductions in sedentary time of the magnitude seen in RCTs (1 h/day/year (Aunger et al. 2018)) were associated with a 2.9 cm/s/year improvement in UWS. The current literature suggests that 10 cm/s improvements in UWS are associated with significant mortality benefit and reductions in incident disability (Abellan Van Kan et al. 2009; Studenski et al. 2011). Currently, interventions to improve physical function tend to focus on resistance training, but less frequently target MVPA during normal daily living and have not targeted sedentary time (Jadczak et al. 2018). It is not clear whether sustained change in physical behaviours is achievable in this population, but our findings support the need for development and evaluation of interventions to achieve this aim and highlight the potential of overlooked parts of the activity spectrum, such as LPA and sedentary time.

### Strengths and limitations

Firstly, we utilised a large population-based sample of older adults (EPIC-Norfolk cohort) aiding the reliability and power of our results. Secondly, physical behaviours were objectively assessed utilising accelerometers rather than self-report questionnaires, which are prone to error and recall bias. Thirdly, individuals were measured at multiple time points, allowing us to do prospective analyses. EPIC-Norfolk participants included in this analysis were healthier than the general population (Craig and Mindell 2007) in terms of blood pressure and BMI likely due to healthy volunteer bias and attrition, which could potentially reduce the generalisability of our results.

Accelerometers, though more precise and less prone to bias than self-report measures, have several notable limitations. They are hip-mounted non-waterproof devices which are not sensitive to certain activities (e.g. standing vs. sitting, upper-body movements, and water-based activities when participants were asked to remove their monitors). The effect of this may be underestimation of PA, and therefore reduced effect sizes. To deal with non-wear misclassifications, we utilised an algorithm with a non-wear time threshold was defined as ≥ 90 min(Mailey et al. 2014). Having examined the stability of each variable, we found that there was enough variability to detect associations with change, albeit less variability for grip strength. There is potential for residual confounding from factors such as change in chronic disease status, as we did not have access to chronic disease status at follow-up.

## Conclusions

Our findings are consistent with a bidirectional relationship between change in objectively assessed physical behaviours and change in physical function related to lower limb physical function (UWS and chair stand speed) rather than upper limb physical function (grip strength) in older adults. We also have found a relationship between baseline physical behaviours and subsequent physical function, and baseline physical function and subsequent physical activity. We therefore have met our objectives in addressing uncertainties about these relationships. Taken together, these observational findings highlight the need for future researchers to explore whether sustained changes in physical behaviours, in particular neglected activity intensities such as LPA, sedentary time and prolonged sedentary bouts, are achievable as one of the potential benefits may be improvements in physical function in later life. This may be important given the role of physical function in healthy ageing and maintenance of independent living.

## Supplementary Information

Below is the link to the electronic supplementary material.

Supplementary file1 (DOCX 49 KB)

## Data Availability

Data from the EPIC-Norfolk study must be requested directly from their data request team by completing a data request form.
