# Supplementary material for: Bidirectional associations of accelerometer-assessed physical activity and sedentary time with physical function among older English adults: the EPIC-Norfolk cohort study
Source: Eur J Ageing. 2022 Oct 21;19(4):1507–17. doi: 10.1007/s10433-022-00733-y (PMC9729509; doi:10.1007/s10433-022-00733-y)
Supplement: Supplementary file 1 — Supplementary file1 (DOCX 49 KB) [file 10433_2022_733_MOESM1_ESM.docx]

**Supplementary Table 1: Baseline Demographic and Clinical Characteristics for Included (n=3188)**  **versus Excluded Participants (n=485)**

| **Characteristics** | | **Percent of Included participants (%)** | **Percent of Excluded participants (%)** |
| --- | --- | --- | --- |
| **Sex** | Male | 45.6 | 39.1 |
|  | Female | 54.4 | 60.9 |
| **Ethnicity** | White | 99.7 | 99.4 |
|  | Other | 0.3 | 0.6 |
| **Occupational Classification** | Professional | 9.0 | 8.4 |
|  | Manager | 42.2 | 44.0 |
|  | Skilled non-manual | 14.7 | 18.2 |
|  | Skilled manual | 21.0 | 17.5 |
|  | Semi-skilled | 10.8 | 10.5 |
|  | Non-skilled | 2.3 | 1.4 |
| **Employed** | No | 75.4 | 79.3 |
|  | Yes | 24.6 | 20.7 |
| **Further Education level** | O-level or lower | 46.0 | 47.3 |
|  | A-level or higher | 54.0 | 52.7 |
| **Smoking Status** | Current | 3.4 | 5.0 |
|  | Former | 46.2 | 44.7 |
|  | Never | 50.4 | 50.3 |
| **History of Chronic Disease** | No | 84.9 | 82.8 |
|  | Yes | 15.1 | 17.2 |
| **Body Mass Index (** kg/m^2^) | <25 | 36.50 | 31.2 |
|  | 25-<30 | 45.8 | 45.7 |
|  | 30-<35 | 13.8 | 17.3 |
|  | ≥35 | 3.9 | 5.8 |

**Supplementary Table 2: Association of baseline physical function with follow-up physical activity and sedentary time**

| **Baseline Physical Function** | **Model Number** | **Follow-up Activity Measures** | | | | | | | | | | | | | | |
| --- | --- | --- | --- | --- | --- | --- | --- | --- | --- | --- | --- | --- | --- | --- | --- | --- |
|  |  | **TPA (cpm)** | | **MVPA (min/day)** | | **LPA (min/day)** | | **Total sedentary time (min/day)** | | **Prolonged sedentary bout time (min/day)** | | ***Sensitivity Analysis*** *(MVPA defined as ≥2020 cpm,. LPA defined as 100-2019 cpm)* | **MVPA (min/day)** | | **LPA (min/day)** | |
|  |  | **β** | **95%CI** | **β** | **95%CI** | **β** | **95%CI** | **β** | **95%CI** | **β** | **95%CI** |  | **β** | **95%CI** | **β** | **95%CI** |
| **Grip strength (per kg) (n=1472)** | 1 | 2.0 | 1.4, 2.6 | 0.8 | 0.5, 1.0 | 0.9 | 1.2, 0.6 | -0.2 | -0.04, -0.6 | -0.8 | -1.4, -0.2 |  | 0.4 | 0.3, 0.5 | 0.6 | 0.2, 1.0 |
|  | 2 | 2.1 | 1.2, 3.0 | 0.8 | 0.5, 1.1 | 0.4 | 0.08, 0.8 | -1.2 | -1.8, -0.5 | -0.6 | -1.4, -0.2 |  | 0.3 | 0.2, 0.5 | 0.9 | 0.3, 1.4 |
|  | 3 | 2.6 | 1.7, 3.4 | 0.9 | 0.6, 1.3 | 0.6 | 0.2, 1.0 | -1.5 | -2.1, -0.9 | -1.0 | -1.8, -0.2 |  | 0.4 | 0.2, 0.5 | 1.1 | 0.5, 1.7 |
| **Usual walking speed (per cm/s) (n=1487)** | 1 | 1.3 | 1.1, 1.5 | 0.4 | 0.3, 0.5 | 0.3 | 0.2, 0.4 | -0.7 | -0.9, -0.6 | -0.8 | -1.0, -0.6 |  | 0.2 | 0.1, 0.2 | 0.6 | 0.4, 0.7 |
|  | 2 | 0.8 | 0.6, 1.0 | 0.2 | 0.2, 0.3 | 0.2 | 0.1, 0.3 | -0.5 | -0.6, -0.3 | -0.4 | -0.7, -0.2 |  | 0.1 | 0.06, 0.1 | 0.4 | 0.2, 0.5 |
|  | 3 | 0.6 | 0.3, 0.8 | 0.2 | 0.09, 0.3 | 0.2 | 0.1, 0.3 | -0.3 | -0.5, -0.1 | -0.2 | -0.4, -0.005 |  | 0.07 | 0.03, 0.1 | 0.2 | 0.08, 0.4 |
| **Chair stand speed (per stand/min) (n=1389)** | 1 | 3.2 | 2.4, 3.9 | 1.1 | 0.8, 1.4 | 0.6 | 0.3, 1.0 | -1.7 | -2.2, -1.2 | -2.0 | -2.7, -1.4 |  | 0.4 | 0.3, 0.6 | 1.3 | 0.8, 1.7 |
|  | 2 | 1.8 | 1.1, 2.5 | 0.6 | 0.3, 0.9 | 0.4 | 0.04, 0.7 | -1.0 | -1.5, -0.5 | -1.1 | -1.7, -0.4 |  | 0.3 | 0.1, 0.4 | 0.7 | 0.3, 1.2 |
|  | 3 | 1.5 | 0.8, 2.3 | 0.5 | 0.3, 0.8 | 0.3 | 0.05, 0.6 | -0.8 | -1.3, -0.3 | -0.9 | -1.5, -0.2 |  | 0.2 | 0.09, 0.3 | 0.6 | 0.1, 1.1 |

*95%CI=95% confidence interval, TPA=total physical activity, MVPA=moderate-to-vigorous activity, LPA=light physical activity, cpm=counts per minute.*

*^a^Model 1 was adjusted for season and wear time at baseline and follow-up.*

*^b^Model 2 was the same as model 1 plus mutually adjusted for age and sex.*

*^c^Model 3 was the same as Model 2 plus mutually adjusted for potential socioeconomic and environmental confounders (job status, smoking status, occupational class, retirement status, BMI, ethnicity, chronic disease status).*

**Supplementary Table 3: Association of baseline physical activity and sedentary time with follow-up physical function**

| **Baseline Activity Measures** | **Model** | **Follow-up Physical Function** | | | | | |
| --- | --- | --- | --- | --- | --- | --- | --- |
|  |  | **Grip strength, kg (n=1433)** | | **Usual walking speed, cm/s (n=1488)** | | **Chair stand speed, stands/min (n=1348)** | |
|  |  | **β** | **95%CI** | **β** | **95%CI** | **β** | **95%CI** |
| **TPA (per 100 count/min)** | 1 | 1.0 | 0.6, 1.5 | 6.8 | 5.8, 7.8 | 1.6 | 1.2, 1.9 |
|  | 2 | 0.1 | -0.2, 0.4 | 4.4 | 2.0, 6.7 | 1.1 | 0.7, 1.4 |
|  | 3 | 0.1 | -0.2, 0.4 | 2.9 | 1.9, 3.9 | 0.8 | 0.5, 1.1 |
| **MVPA (per hour/day)** | 1 | 1.8 | 1.2, 2.4 | 12.0 | 8.0, 15.0 | 1.8 | 1.2, 2.4 |
|  | *2* | 0.2 | -0.2, 0.6 | 5.4 | 4.2, 6.0 | 1.2 | 0.6, 1.8 |
|  | *3* | 0.2 | -0.3, 0.6 | 4.2 | 2.4, 5.4 | 1.2 | 0.4, 2.0 |
| **LPA (per hour/day)** | 1 | -2.4 | -3.0, -1.8 | 4.2 | 2.6, 5.4 | 1.2 | 0.5, 2.1 |
|  | 2 | -0.04 | -0.5, 0.4 | 3.0 | 1.8, 4.2 | 0.6 | 0.4, 0.8 |
|  | 3 | -0.01 | -0.4, 0.4 | 1.8 | 0.6, 3.6 | 0.7 | 0.2, 1.2 |
| **Total sedentary time (per hour/day)** | 1 | 0.6 | 0.1, 1.0 | -5.0 | -6.0, -4.1 | -1.2 | -1.4, -0.8 |
|  | 2 | -0.07 | -0.4, 0.2 | -3.4 | -4.3, -2.5 | -0.9 | -1.2, -0.6 |
|  | 3 | -0.07 | -0.4, 0.2 | -2.3 | -3.2, -1.4 | -0.7 | -1.0, -0.4 |
| **Prolonged sedentary bout time (per hour/day)** | 1 | 0.7 | 0.3, 1.0 | -4.4 | -5.2, -3.6 | -1.0 | -1.3, -0.8 |
|  | 2 | 0.1 | -0.1, 0.4 | -2.9 | -3.7, -2.1 | -0.8 | -1.1, -0.5 |
|  | 3 | 0.1 | -0.1, 0.4 | -1.7 | -2.6, -1.0 | -0.6 | -0.8, -0.3 |
| ***Sensitivity Analysis (****MVPA defined as ≥2020cpm, LPA defined as 100-2019 cpm)* | | | | | | | |
| **MVPA (per hour/day)** | 1 | 5.4 | 4.8, 6.0 | 18.0 | 12.0, 24.0 | 4.2 | 3.0, 5.4 |
|  | *2* | 0.06 | -1.2, 1.2 | 12.0 | 6.0, 18.0 | 2.4 | 1.2, 3.6 |
|  | *3* | -0.01 | -1.2, 1.2 | 6.0 | 2.4, 12.0 | 1.2 | 0.2, 3.0 |
| **LPA ( per hour/day)** | 1 | -1.0 | -1.1, -0.6 | 4.4 | 3.4, 5.4 | 1.0 | 0.7, 1.4 |
|  | 2 | 0.07 | -0.2, 0.4 | 3.0 | 2.0, 4.0 | 0.8 | 0.5, 1.1 |
|  | 3 | 0.09 | -0.2, 0.4 | 2.2 | 1.2, 3.1 | 0.7 | 0.3, 1.0 |

*Given the small magnitude of B, units are displayed here as per hour/day for LPA and ST and per 100 counts/min for TPA.*

*95%CI=95% confidence interval, TPA=total physical activity, MVPA=moderate-to-vigorous activity, LPA=light physical activity.*

*^a^Model 1 was adjusted for season and wear time at baseline and follow-up.*

*^b^Model 2 was the same as model 1 plus mutually adjusted for age and sex.*

*^c^Model 3 was the same as Model 2 plus mutually adjusted for potential socioeconomic and environmental confounders (job status, smoking status, occupational class, retirement status, BMI, ethnicity, chronic disease status).*

**Supplementary Table 4: Association of change in physical function with change in physical behaviours**

| **Change in Physical Function** | **Model Number** | **Change in Physical Behaviours** | | | | | | | | | | | | | | |
| --- | --- | --- | --- | --- | --- | --- | --- | --- | --- | --- | --- | --- | --- | --- | --- | --- |
|  |  | **TPA (cpm/year)** | | **MVPA (min/day/year)** | | **LPA (min/day/year)** | | **Total sedentary time (min/day/year)** | | **Prolonged sedentary bout time (min/day/year)** | | ***Sensitivity Analysis*** *(MVPA defined as ≥2020 cpm,. LPA defined as 100-2019 cpm)* | **MVPA (min/day/year)** | | **LPA (min/day/year)** | |
|  |  | **β** | **95%CI** | **β** | **95%CI** | **β** | **95%CI** | **β** | **95%CI** | **β** | **95%CI** |  | **β** | **95%CI** | **β** | **95%CI** |
| **Grip strength, (per kg/year) (n=1408)** | **1** | 0.8 | 0.02, 1.5 | 0.2 | -0.1, 0.4 | 0.1 | -0.3, 0.5 | -0.04 | -0.6, 0.5 | -0.2 | -0.8, 0.5 |  | 0.2 | 0.009, 0.3 | 0.09 | -0.4, 0.06 |
|  | **2** | 0.6 | -0.2, 1.3 | 0.05 | -0.2, 0.4 | 0.2 | -0.2, 0.6 | -0.009 | -0.5, 0.5 | -0.03 | -0.7, 0.7 |  | 0.1 | -0.04, 0.3 | 0.1 | -0.4, 0.6 |
|  | **3** | 0.4 | -0.4, 1.2 | 0.02 | -0.3, 0.3 | 0.09 | -0.3, 0.5 | 0.1 | -0.4, 0.6 | 0.1 | -0.6, 0.8 |  | 0.09 | -0.06, 0.2 | 0.02 | -0.5, 0.5 |
| **Usual walking speed, (per cm/s/year) (n=1477)** | **1** | 0.8 | 0.6, 1.0 | 0.3 | 0.2, 0.3 | 0.4 | 0.2, 0.5 | -0.6 | -0.7, -0.4 | -0.6 | -0.8, -0.4 |  | 0.1 | 0.07, 0.2 | 0.5 | 0.3, 0.6 |
|  | **2** | 0.7 | 0.4, 0.9 | 0.2 | 0.1, 0.3 | 0.3 | 0.2, 0.4 | -0.5 | -0.6, -0.3 | -0.5 | -0.6, -0.3 |  | 0.08 | 0.04, 0.1 | 0.4 | 0.3, 0.5 |
|  | **3** | 0.6 | 0.4, 0.8 | 0.2 | 0.1, 0.3 | 0.3 | 0.1, 0.4 | -0.4 | -0.6, -0.3 | -0.4 | -0.6, -0.2 |  | 0.08 | 0.03, 0.1 | 0.4 | 0.2, 0.5 |
| **Chair stand speed (per stands/min/year) (n=1286)** | **1** | 1.4 | 0.7, 2.1 | 0.4 | 0.1, 0.6 | -0.02 | -0.3, 0.3 | -0.5 | -0.9, -0.07 | -0.8 | -1.4, -0.3 |  | 0.3 | 0.1, 0.4 | 0.08 | -0.4, 0.5 |
|  | **2** | 1.1 | 0.5, 1.8 | 0.3 | 0.03, 0.5 | -0.03 | -0.4, 0.3 | -0.4 | -0.8, 0.03 | -0.8 | -1.3, -0.2 |  | 0.2 | 0.08, 0.3 | 0.0004 | -0.4, 0.4 |
|  | **3** | 1.0 | 0.4, 1.7 | 0.3 | 0.0007, 0.5 | -0.1 | -0.4, 0.2 | -0.3 | -0.8, 0.1 | -0.7 | -1.2, -0.1 |  | 0.2 | 0.06, 0.3 | -0.08 | -0.5, 0.3 |

*95%CI=95% confidence interval, TPA=total physical activity, MVPA=moderate-to-vigorous activity, LPA=light physical activity.*

*^a^Model 1 was adjusted for season and wear time at baseline and follow-up, baseline activity measure and baseline physical function measure.*

*^b^Model 2 was the same as model 1 plus mutually adjusted for age and sex.*

*^c^Model 3 was the same as Model 2 plus mutually adjusted for potential socioeconomic and environmental confounders (job status, smoking status, occupational class, retirement status, BMI, ethnicity, chronic disease status)*

**Supplementary Table 5: Association of change in physical behaviours with change in physical function**

| **Change in Physical Behaviours** | **Model Number** | **Change in Physical Function** | | | | | |
| --- | --- | --- | --- | --- | --- | --- | --- |
|  |  | **Grip strength, kg/year (n=1408)** | | **Usual walking speed, cm/s/year (n=1477)** | | **Chair stand speed, stands/min/year (n=1286)** | |
|  |  | **β** | **95%CI** | **β** | **95%CI** | **β** | **95%CI** |
| **TPA(per 100 cpm/year)** | 1 | 0.4 | 0.01, 0.7 | 4.4 | 3.2, 5.5 | 0.9 | 0.5, 1.3 |
|  | 2 | 0.3 | -0.1, 0.6 | 3.4 | 2.2, 4.6 | 0.8 | 0.3, 0.1 |
|  | 3 | 0.2 | -0.2, 0.5 | 3.1 | 0.1, 4.2 | 0.7 | 0.2, 1.1 |
| **MVPA (per hour/day/year)** | 1 | 0.3 | -0.3, 0.6 | 6.0 | 3.6, 8.0 | 1.2 | 0.4, 1.8 |
|  | 2 | 0.1 | -0.5, 1.2 | 4.2 | 2.4, 6.0 | 0.6 | 0.6, 1.2 |
|  | 3 | 0.04 | -0.6, 0.6 | 3.6 | 1.8, 5.4 | 0.6 | 0.002, 1.2 |
| **LPA (per hour/day/year)** | 1 | 0.1 | -0.3, 0.5 | 4.2 | 3, 5.4 | -0.03 | -0.6, 0.5 |
|  | 2 | 0.2 | -0.2, 0.6 | 3.6 | 2.4, 4.8 | -0.06 | -0.6, 0.5 |
|  | 3 | 0.01 | -0.3, 0.3 | 2.5 | 1.4, 3.5 | -0.08 | -0.5, 0.3 |
| **Total sedentary time (per hour/day/year)** | 1 | -0.02 | -3.4, 0.3 | -4.1 | -5.1, -3.1 | -0.5 | -0.9, -0.07 |
|  | 2 | -0.005 | -0.3, 0.3 | -3.4 | -4.4, -2.3 | -0.4 | -0.8, 0.03 |
|  | 3 | 0.06 | -0.3, 0.4 | -2.9 | -4.0, -1.9 | -0.3 | -0.7, 0.1 |
| **Prolonged sedentary bout time (per hour/day/year)** | 1 | -0.07 | -0.3, 0.2 | -2.5 | -3.3, -1.7 | -0.6 | -0.9, -0.2 |
|  | 2 | -0.01 | -0.3, 0.2 | -1.9 | -2.7, -1.1 | -0.5 | -0.8, -0.2 |
|  | 3 | 0.04 | -0.2, 0.3 | -1.6 | -2.4, -0.8 | -0.4 | -0.8, -0.1 |
| ***Sensitivity Analysis (****MVPA defined as ≥2020cpm, LPA defined as 100-2019 cpm)* | | | | | | | |
| **MVPA (per hour/day/year)** | 1 | 1.2 | 0.6, 2.4 | 12.0 | 6.0, 14.0 | 3.0 | 1.2, 4.2 |
|  | 2 | 0.6 | -0.3, 1.8 | 6.0 | 3.6, 12.0 | 2.4 | 0.6, 3.6 |
|  | 3 | 0.6 | -0.5, 1.8 | 6.0 | 2.4, 12.0 | 1.8 | 0.6, 3.6 |
| **LPA (per hour/day/year)** | 1 | 0.06 | -0.3, 0.4 | 3.5 | 2.5, 4.6 | 0.7 | -0.4, 0.5 |
|  | 2 | 0.06 | -0.3, 0.4 | 2.8 | 1.8, 3.9 | 0.04 | -0.4, 0.4 |
|  | 3 | 0.01 | -0.3, 0.3 | 2.5 | 1.4, 3.5 | -0.08 | -0.5, 0.3 |

*95%CI=95% confidence interval, TPA=total physical activity, MVPA=moderate-to-vigorous activity, LPA=light physical activity.*

*^a^Model 1 was adjusted for season and wear time at baseline and follow-up, baseline activity measure and baseline physical function*

*^b^Model 2 was the same as model 1 plus mutually adjusted for age and sex.*

*^c^Model 3 was the same as Model 2 plus mutually adjusted for potential socioeconomic and environmental confounders (job status, smoking status, occupational class, retirement status, BMI, ethnicity, chronic disease status).*

**Supplementary Table 6: Correlation of baseline and follow-up values (R) of Physical Behaviour and Physical Function**

| **Variable** | **Correlation of baseline and follow-up values ( R)** |
| --- | --- |
| UWS | 0.58 |
| Grip strength | 0.83 |
| Chair stand speed | 0.54 |
| TPA | 0.68 |
| MVPA | 0.65 |
| LPA | 0.61 |
| Total sedentary time | 0.66 |
| Prolonged sedentary bout | 0.65 |
